# Typhoid Test in Nurses and Physicians

**Published:** 1917-04

**Authors:** 


**Typhoid Test in Nurses and Physicians.** F. P. Gay and A. R. Lamb, Jour. of Lab. and Clin. Med. Jan., discuss the results of the dermal test originally described by Gay and Force.

12 persons, giving distinct history of typhoid 2-22 years previously, 9 positive, 75%

29 vaccinated within 6 months, 19 positive, 65.5%

50 vaccinated 6 months to 1 year previously, 32 positive, 64%

24 vaccinated 1-1½ years previously, 18 positive, 75%

13 vaccinated 2-2½ years previously, 7 positive, 53.8%

5 vaccinated 3-5 years previously, 2 positive, 40%. These had had various vaccines, the others N. Y. Board of Health vaccine.

21 persons who had not had typhoid nor been vaccinated, 3 positive, 14.28%

